# Two new species of the genus *Camptoscaphiella* from Yunnan, China (Araneae, Oonopidae)

**DOI:** 10.3897/zookeys.1052.66743

**Published:** 2021-07-30

**Authors:** Ying Huang, Dongju Bian, Yanfeng Tong, Shuqiang Li

**Affiliations:** 1 Life Science College, Shenyang Normal University, Shenyang 110034, China Shenyang Normal University Shenyang China; 2 CAS Key Laboratory of Forest Ecology and Management, Institute of Applied Ecology, Shenyang 110016, China Institute of Applied Ecology Shenyang China; 3 Institute of Zoology, Chinese Academy of Sciences, Beijing 100101, China Institute of Zoology, Chinese Academy of Sciences Beijing China

**Keywords:** Asia, Gamasomorphinae, goblin spiders, morphology, taxonomy

## Abstract

Two new species of the genus *Camptoscaphiella* Caporiacco, 1934 are described from Yunnan, China, i.e., *C. changxu* Tong & Li, **sp. nov.** (♂♀) and *C. linyejiei* Tong & Li, **sp. nov.** (♂♀). *Camptoscaphiella changxu* Tong & Li, **sp. nov.** is characterized by the long, strongly-curved setae between male paturons, which is unknown in other oonopids and in any other spiders. *Camptoscaphiella linyejiei* Tong & Li, **sp. nov.** is the third blind species of this genus in the world. Morphological descriptions and photographic illustrations of the two new species are given.

## Introduction

*Camptoscaphiella* Caporiacco, 1934, is a small genus of oonopid spiders that currently contains 18 species (Li 2020; WSC 2021). It is distributed in montane tropical and subtropical regions in Asia, mostly within the Himalayan Plateau (Baehr and Ubick 2010). This genus can be recognized by the remarkable morphology of the male palp, which has an extremely large, club-shaped palpal patella and a bulb that is not fused with the cymbium, and the first two pairs of legs which have extremely long spines with the tibiae bearing 4 pairs of spines and the metatarsi bearing 2 pairs of spines (Baehr and Harvey 2013).

*Camptoscaphiella* is still poorly studied. Currently three species of this genus are recorded in Yunnan, China, i.e., *C. paquini* Ubick, 2010, *C. sinensis* Deeleman-Reinhold, 1995 and *C. tuberans* Tong & Li, 2007, and only one species, *C. schwendingeri* Baehr, 2010 is recorded in Thailand (Deeleman-Reinhold 1995; Tong and Li 2007; Baehr and Ubick 2010). There is no species recorded in the adjacent areas of south of Yunnan, i.e., Laos, Myanmar and Vietnam. In this paper two new *Camptoscaphiella* species, *C. changxu* Tong & Li, sp. nov. and *C. linyejiei* Tong & Li, sp. nov. collected from Yunnan, are described and illustrated.

## Materials and methods

The specimens were examined using a Leica M205C stereomicroscope. Details were studied under an Olympus BX51 compound microscope. Photos were made with a Canon EOS 550D zoom digital camera (18 megapixels) mounted on an Olympus BX51 compound microscope. Vulvae were cleared in lactic acid. For scanning electron microscopy (SEM), specimens were air-dried, sputter-coated using IXRF SYSTEMS, and imaged with a Hitachi TM3030 SEM. All measurements were taken using an Olympus BX51 compound microscope and are in millimeters. The type material is deposited in Shenyang Normal University (**SYNU**) in Shenyang, China.

The following abbreviations are used in the text and figures: a = apodemes; ALE = anterior lateral eyes; cd = copulatory duct; dp = dorsal process; PLE = posterior lateral eyes; PME = posterior median eyes; po = pore; pp = prolateral process; rp = retrolateral process; ss = star-shaped structure; ssc = stick-shaped sclerite; trs = transverse sclerites; vp = ventral process; vsp = ventral small process; wa = wing-shaped appendices; XTBG = Xishuangbanna Tropical Botanical Garden.

## Taxonomy

### Family Oonopidae Simon, 1890

#### Genus Camptoscaphiella

Taxon classificationAnimaliaAraneaeOonopidae

Caporiacco, 1934

D2165190-77CD-5249-8E8F-85CB580FDB93

##### Type species.

*Camptoscaphiella fulva* Caporiacco, 1934, by monotypy.

##### Diagnosis.

Males of this genus can easily be separated from all other oonopid genera by the heart-shaped sternum with conical projection on the anterolateral corners, spination of the first two legs (tibia I and II with four pairs of long spines, and metatarsus I and II with two pairs of long spines); the extremely large, club-shaped palpal patella; and a cymbium that is not fused with the bulb. Females of this genus are similar to those of *Ischnothyreus* Simon, 1893, but can be separated by lacking the distinct, darkly sclerotized, strongly winding duct and uniquely shaped atrium (revised from Baehr and Harvey 2013).

##### Distribution.

China (Yunnan), New Caledonia, South Asia (Bhutan, India, Pakistan, Sri Lanka), Southeast Asia (Thailand).

#### Camptoscaphiella changxu

Taxon classificationAnimaliaAraneaeOonopidae

Tong & Li sp. nov.

446C9D4F-BC5D-5E81-A297-F595E9D523EA

http://zoobank.org/4FA56EE3-F9C4-4B6F-8AD0-0E2C0A6FF85C

Figures 1 , 2 , 3

##### Type material.

***Holotype*** ♂ China, Yunnan, Menglun, XTBG, *Paramichelia baillonii* plantation; 21°53.823'N, 101°17.072'E; 613 m; pitfall traps; 1–15 May 2007; Guo Zheng leg. (SYNU-481). ***Paratypes*** [same data as holotype except where indicated] 1♂ (SYNU-482); 1♂ (SYNU-494); 1♂1♀, pitfall traps, 16–31 June 2007 (SYNU-483–484); 1♂, pitfall traps, 1–9 Dec. 2006 (SYNU-485); 1♂, pitfall traps, 1–9 Dec. 2006 (SYNU-486); 1♀ (SYNU-487); 1♂1♀, XTBG, Secondary tropical montane evergreen broad-leaved forest, 21°54.813'N, 101°12.634'E, 876 m, pitfall traps, 16–24 Sep. 2006, Guo Zheng leg. (SYNU-488–489); 1♂1♀, pitfall traps, 1–15 July 2007 (SYNU-490–491); 1♂1♀, pitfall traps, 16–31 June 2007 (SYNU-492–493).

##### Diagnosis.

**Figure 1.**
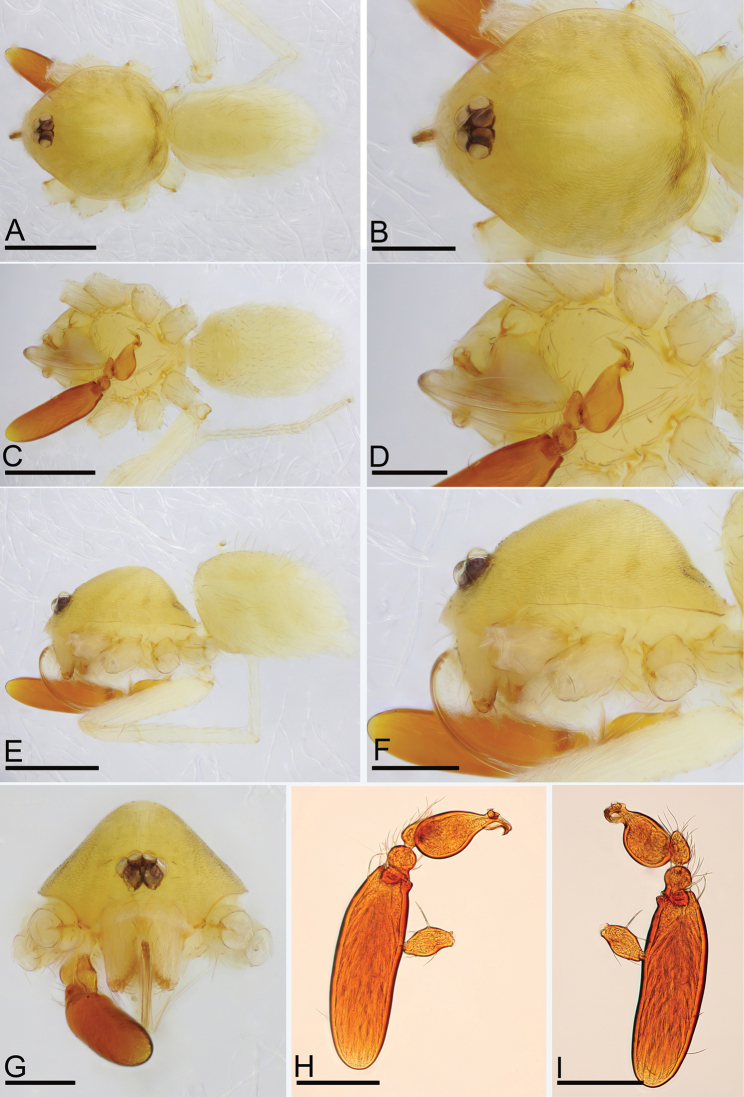
*Camptoscaphiella changxu* sp. nov., male holotype (SYNU-481) **A, C, E** habitus in dorsal, ventral, and lateral views **B, D, F, G** prosoma in dorsal, ventral, lateral, and anterior views **H, I** left palp in prolateral and retrolateral views. Scale bars: 0.4 mm (**A, C, E**); 0.2 mm (**B, D, F–I**).

**Figure 2.**
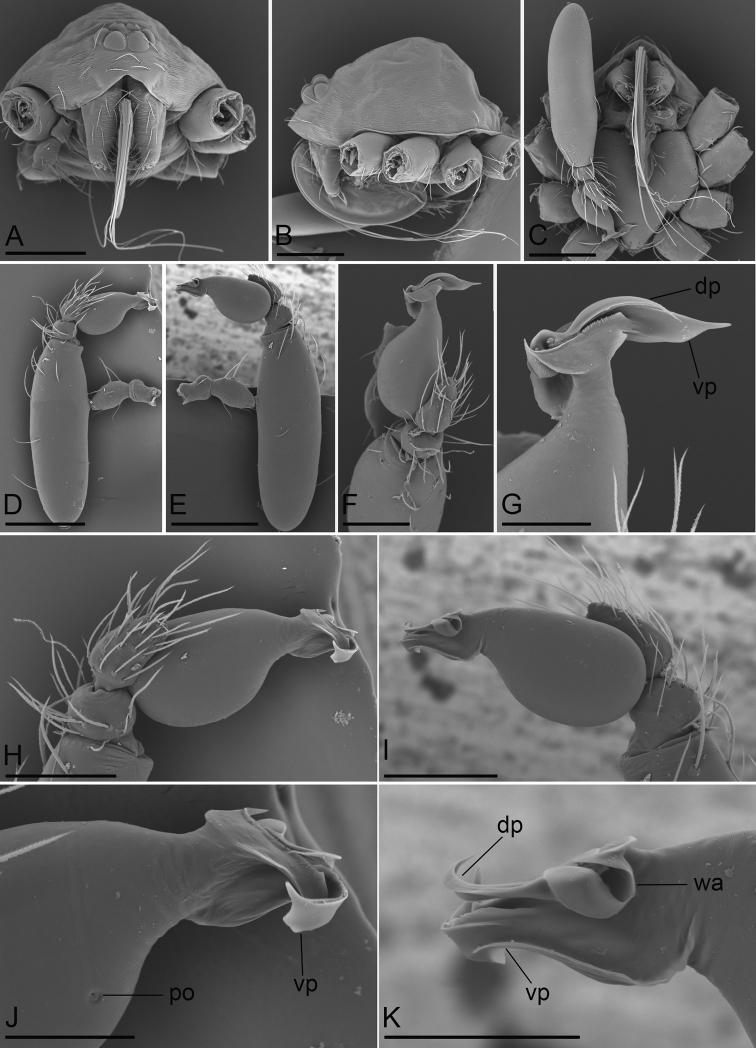
*Camptoscaphiella changxu* sp. nov., male holotype (SYNU-481), SEM**A–C** prosoma in anterior, lateral, and ventral views **D–F** left palp, prolateral, retrolateral, and dorsal views **G, J, K** distal part of bulb, dorsal, prolateral, and retrolateral views **H, I** left palpal bulb, prolateral and retrolateral views. Abbreviations: dp = dorsal process; po = pore; vp = ventral process; wa = wing-shaped appendices. Scale bars: 0.2 mm (**A–E**); 0.1 mm (**F, H, I**); 0.05 mm (**G, J, K**).

**Figure 3.**
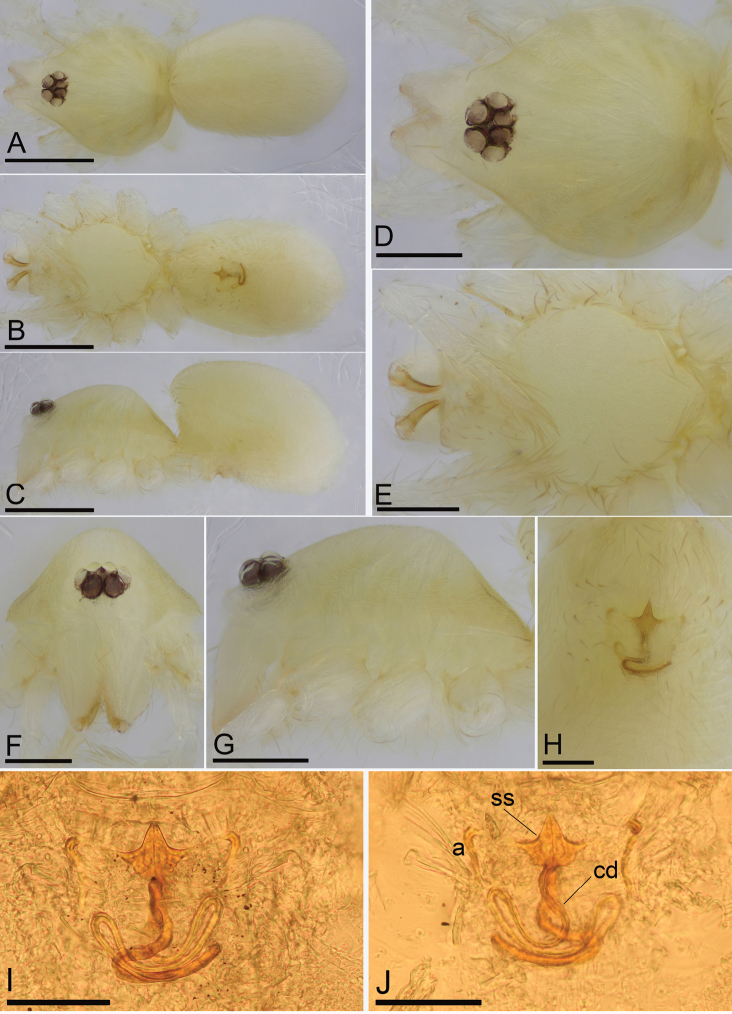
*Camptoscaphiella changxu* sp. nov., female paratype (SYNU-484) **A–C** habitus in dorsal, ventral, and lateral views **D–G** prosoma in dorsal, ventral, anterior, and lateral views **H** epigastric region, ventral view **I, J** endogyne (cleared in lactic acid), ventral and dorsal views. Abbreviations: a= apodemes; cd = copulatory duct; ss = star-shaped structure. Scale bars: 0.4 mm (**A–C**); 0.2 mm (**D–G**); 0.1 mm (**H–J**).

This new species is similar to *C. schwendingeri* Baehr, 2010 (female unknown), but can be distinguished by the long, strongly-curved setae between the cheliceral paturons (Figs 1F, G, 2A–C) and a uniformly coloured carapace (vs setae absent and the carapace with a longitudinal brown stripe (Baehr and Ubick 2010: figs 327–331)). Females of this new species can be distinguished from congeners by the star-shaped structure of the endogyne and the strongly-curved copulatory duct (Fig. 3H–J).

##### Description.

**Male (holotype)**: ***Body***: uniformly colored, yellow; habitus as in Fig. 1A, C, E; length 1.29. ***Carapace*** (Fig. 1B, F): 0.65 long, 0.59 wide; pars cephalica strongly elevated in lateral view, surface of elevated portion and sides of pars cephalica finely reticulate. ***Eyes*** (Fig. 1B, G): ALE 0.057; PME 0.049; PLE 0.049; ALE circular, PME oval, PLE oval; posterior eye row procurved from both above and front; ALE separated by less than one radius. ***Clypeus*** (Fig. 1F, G): margin unmodified, straight in front view, sloping forward in lateral view. ***Mouthparts*** (Figs 1F, G, 2A–C): with several long, strongly-curved setae between paturons. ***Sternum*** (Fig. 1D): as long as wide, pale orange, surface finely reticulate. ***Abdomen*** (Fig. 1A, C, E): 0.67 long, 0.41 wide; oval, scuta pale orange; dorsal scutum covering about 2⁄3 of abdomen length, between 1/2 and 3⁄4 of abdomen width, not fused to epigastric scutum; postgastric scutum small, just near epigastric furrow. ***Legs***: pale orange. ***Palp*** (Figs 1H, I, 2D–K): reddish-brown; patella extremely long club-shaped, about 4.3 times of the femur length, and 2.1 times of the bulb length; cymbium narrow in dorsal view; distal part of bulb with a broad ventral process (vp) and a narrow dorsal process (dp), the dorsal one with basally wing-shaped appendices (wa).

**Female (SYNU-484)**: ***Body***: habitus as in Fig. 3A–C; length 1.37. ***Carapace***: 0.62 long, 0.57 wide. ***Eyes***: ALE 0.065; PME 0.049; PLE 0.057. ***Mouthparts***: chelicerae unmodified. ***Abdomen***: 0.79 long, 0.52 wide. ***Epigastric area*** (Fig. 3H, I): surface without external features. ***Endogyne*** (Fig. 3J): with an anterior star-shaped structure (ss); copulatory duct (cd) very long, strongly curved; apodemes thread-shaped (a).

##### Etymology.

The specific name is derived from Chinese pinyin, “changxu”, which means “macrochaeta”, referring to the long, curved setae between male cheliceral paturons; noun in apposition.

##### Comments.

The male chelicerae are unmodified in species considered to belong to the genus (Baehr and Ubick 2010). The long, strongly-curved setae between male cheliceral paturon of this species are unique in this genus, even in the entire order.

##### Distribution.

Known only from the type locality.

#### Camptoscaphiella linyejiei

Taxon classificationAnimaliaAraneaeOonopidae

Tong & Li sp. nov.

E5638B53-20AE-55B1-A162-23BF1CE149E5

http://zoobank.org/FB8CB22C-F158-4B92-8C8B-AA763217B744

Figures 4 , 5 , 6

##### Type material.

***Holotype*** ♂ China, Yunnan, Baoshan City, Longling County, Datianba Village, Xianren Cave; 24°358.09'N, 99°037.93'E; 3 Oct. 2020; Yejie Lin Leg. (SYNU-479). ***Paratype*** 1♀: same data as holotype (SYNU-480).

##### Diagnosis.

**Figure 4.**
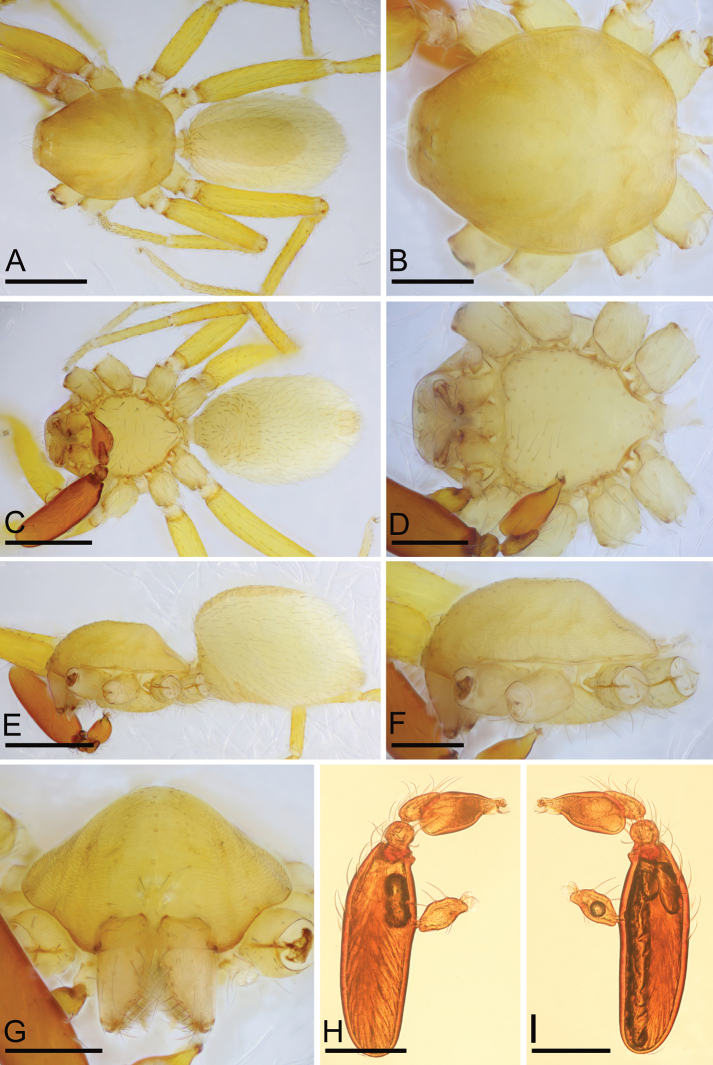
*Camptoscaphiella linyejiei* sp. nov., male holotype (SYNU-479) **A, C, E** habitus in dorsal, ventral, and lateral views **B, D, F, G** prosoma in dorsal, ventral, lateral, and anterior views **H, I** left palp in prolateral and retrolateral views. Scale bars: 0.4 mm (**A, C, E**); 0.2 mm (**B, D, F–I**).

**Figure 5.**
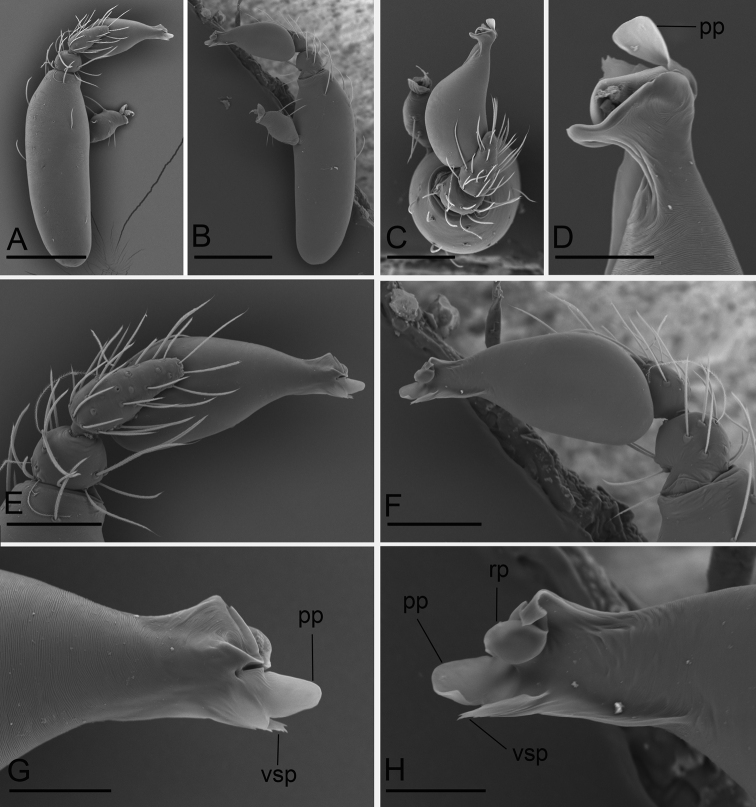
*Camptoscaphiella linyejiei* sp. nov., male holotype (SYNU-479), SEM**A–C** left palp, prolateral, retrolateral, and dorsal views **D, G, H** distal part of bulb, dorsal, prolateral, and retrolateral views **E, F** left palpal bulb, prolateral and retrolateral views. Abbreviations: pp = prolateral process; rp = retrolateral process; vsp = ventral small process. Scale bars: 0.2 mm (**A, B**); 0.1 mm (**C, E, F**); 0.03 mm (**D, G, H**).

This new species is similar to *C. sinensis*, but can be distinguished by the flat carapace (Fig. 4F), the presence of dorsal and ventral abdominal scuta (Fig. 4A, C, E), the unmodified cymbium (Fig. 5E), and the short processes of tip of the bulb (Fig. 5G, H). *Camptoscaphiella sinensis* has the highest point of carapace at posterior 2/3, the abdomen lacking scuta, the tip of the cymbium with a pair of enlarged tubular setae, and the tip of bulb with elongated processes (Deeleman-Reinhold 1995: figs 1–5).

##### Description.

**Male (holotype)**: ***Body***: uniformly yellow; habitus as in Fig. 4A, C, E; length 1.53. ***Carapace*** (Fig. 4B, F): 0.71 long, 0.56 wide; pars cephalica slightly elevated in lateral view, surface of elevated portion and sides of pars cephalica finely reticulated. ***Eyes*** (Fig. 4B, G): reduced, with only remnants. ***Clypeus*** (Fig. 4F, G): margin unmodified, straight in anterior view, sloping forward in lateral view. ***Mouthparts*** (Fig. 4D, G): chelicerae slightly divergent, anterior-median part of the endites strongly sclerotized. ***Sternum*** (Fig. 4D): pale orange, surface finely reticulated. ***Abdomen*** (Fig. 4A, C, E): 0.77 long, 0.48 wide; dorsal scutum covering 2⁄3 of abdomen length, 1⁄2 of abdomen width. ***Legs***: pale orange. ***Palp*** (Figs 4H, I, 5A–H): reddish-brown; patella extremely long club-shaped, about 4.3 times of the femur length, and 2.2 times of the bulb length; cymbium narrow in dorsal view; distal part of bulb with a rectangular prolateral process (pp), a round retrolateral process (rp) and a small ventral process (vsp).

**Figure 6.**
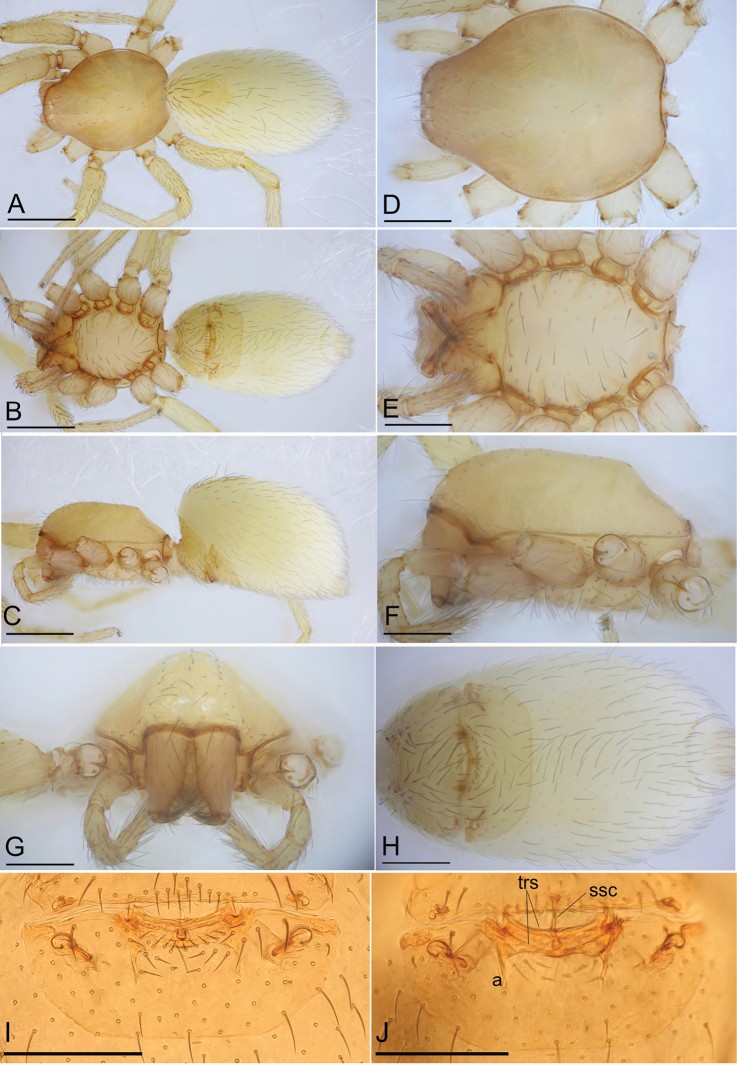
*Camptoscaphiella linyejiei* sp. nov., female paratype (SYNU-480) **A–C** habitus in dorsal, ventral, and lateral views **D–G** prosoma in dorsal, ventral, lateral, and anterior views **H** abdomen, ventral view **I, J** endogyne (cleared in lactic acid), ventral and dorsal views. Abbreviations: a = apodemes; ssc = stick-shaped sclerite; trs = transverse sclerites. Scale bars: 0.4 mm (**A–C**); 0.2 mm (**D–J**).

**Female (SYNU-480)**: ***Body***: habitus as in Fig. 6A–C; length 1.75. ***Carapace***: 0.76 long, 0.61 wide. ***Abdomen***: 1.04 long, 0.63 wide. ***Epigastric area*** (Fig. 6H, I): surface without external features. ***Endogyne*** (Fig. 6J): with two transverse sclerites (trs) and a median stick-shaped sclerite (ssc); apodemes (a) thread-shaped.

##### Etymology.

The specific name is named after Mr Yejie Lin, the collector of the type specimens; noun (name) in genitive case.

##### Distribution.

Known only from the type locality.

## Supplementary Material

XML Treatment for Camptoscaphiella

XML Treatment for Camptoscaphiella changxu

XML Treatment for Camptoscaphiella linyejiei
